# Expression of the Sweet Potato MYB Transcription Factor *IbMYB48* Confers Salt and Drought Tolerance in Arabidopsis

**DOI:** 10.3390/genes13101883

**Published:** 2022-10-17

**Authors:** Hongyuan Zhao, Haoqiang Zhao, Yuanfeng Hu, Shanshan Zhang, Shaozhen He, Huan Zhang, Ning Zhao, Qingchang Liu, Shaopei Gao, Hong Zhai

**Affiliations:** 1 Key Laboratory of Sweet Potato Biology and Biotechnology, Ministry of Agriculture and Rural Affairs/Beijing Key Laboratory of Crop Genetic Improvement/Laboratory of Crop Heterosis and Utilization, Ministry of Education, College of Agronomy & Biotechnology, China Agricultural University, Beijing 100193, China; 2 Sanya Institute, China Agricultural University, Sanya 572025, China

**Keywords:** sweet potato, *IbMYB48*, MYB transcription factor, salt and drought tolerance

## Abstract

Sweet potato (*Ipomoea batatas* (L.) Lam) is one of the most crucial food crops widely cultivated worldwide. In plants, MYB transcription factors play crucial roles in plant growth, defense regulation, and stress resistance. However, the regulatory mechanism of MYBs in salt and drought response remain poorly studied in sweet potato. By screening a transcriptome database for differentially expressed genes between the sweet potato variety Jingshu 6 and its mutant JS6-5 with high anthocyanin and increased tolerance to salt and drought stresses, we identified a R2R3-MYB gene *IbMYB48*, for which expression was induced by PEG6000, NaCl, abscisic acid (ABA), methyl jasmonic acid (MeJA), salicylic acid (SA) and H_2_O_2_. Particle-mediated transient transformation of onion epidermal cells showed IbMYB48 is localized in the nucleus. Transactivation activity assay in yeast cells revealed that *IbMYB48* has transactivation activity, and its active domain is located in the carboxyl (C)-terminal region. Furthermore, expression of *IbMYB48* confers enhanced tolerance to salt and drought stresses in transgenic Arabidopsis. The contents of endogenous ABA, JA, and proline in transgenic lines were higher than control, and the activity of superoxide dismutase (SOD) was significantly increased under salt and drought stress conditions. By contrast, the accumulation of malondialdehyde (MDA) and H_2_O_2_ were lower. Moreover, genes encoding enzymes involved in ABA biosynthetic pathway, JA biosynthesis and signaling pathway, and reactive oxygen species (ROS) scavenging system were significantly up-regulated in transgenic Arabidopsis under salt or drought stress. Altogether, these results suggest *IbMYB48* may be a candidate gene for improvement of abiotic stress tolerance.

## 1. Introduction

Plants are all the products of eons of evolution from primal organisms in response to abiotic and biotic stress conditions [1]. Salt and drought stresses are major abiotic stress in plant agriculture worldwide, because these unfavorable environmental factors will negatively affect the normal growth and development of crops [2,3,4]. Therefore, a more detailed reaction processes are needed to understand the mechanism of the stress response [5]. Plants have evolved a series of mechanisms to respond and adapt to adverse environment [6]. The regulatory networks consist of stress sensors, a network of protein–protein reactions, transcription factors and promoters, and eventually the output proteins or metabolites [2]. For instance, ABA plays a key role in the plant’s response to abiotic stress, such as drought, salinity, cold, and hypoxia [7]. Stress responses primarily include transcriptional regulation of gene expression, which depends on the interaction of transcription factors with *cis*-regulatory sequences [2]. Numerous signaling proteins including transcription factors, and protein kinases, play signal transduction roles during plant adaptation to abiotic stress, when plants are involved in ranging from stress signal perception to stress-responsive gene expression [8]. Transcription factors (TFs) are generally the earliest response to abiotic stress, which act as significant coordinators of signal transduction [9]. Core sets of transcription factor family genes are distinctively expressed in response to increased abiotic stress, including bZIP, WRKY, AP2/ERF, MYB, bHLH, and NAC families [10].

Among the different TFs, MYBs, as one of the most widely distributed transcriptions factor families in plants are engaged in plant development and response to stresses by binding to *cis*-elements in promoter regions of target genes [11]. MYB protein are characterized by a highly conserved DNA-binding domain of approximately 50 amino acids, which usually comprises up to four imperfect amino acid sequence repeats [12]. Depending on number of consecutive repeats, MYB protein can be divided into four classes: 1R-MYB, R2R3-MYB, 3R-MYB and 4R-MYB [12]. The majority of plant *MYB* genes encode a protein of the R2R3-MYB subfamily, by the absence of the sequences encoding the R1 repeat and following expansion of the gene family [13]. The functions of several R2R3-MYB TFs have been proven in different plants using genetic and molecular methods. MYBs play an important role in abiotic and biotic stresses [11,12,13]. In Arabidopsis, such as *AtMYB2*, *AtMYB15*, *AtMYB44* and *AtMYB60* regulate ABA and abiotic stresses [14,15,16,17]. *AtMYB15* is responsible for cold-regulation of *CBF* genes and in freezing tolerance, mutation of *AtMYB15* shows increased tolerance to freezing stress in contrast to overexpression of *AtMYB15* [16]. *AtMYB44*, a R2R3-MYB gene of Arabidopsis, is activated under various abiotic stress, such as dehydration, low temperature and salinity [17]. Correspondingly, *AtMYB44* overexpression transgenic plants obviously enhance tolerance to salt and drought stress compared to wild-type (WT) plants [17]. Overexpression of wheat *TaMYB19* and *TaMYB30-B* improve drought stress tolerance in transgenic Arabidopsis [18,19]. Overexpression of soybean *GmMYB76* or *GmMYB177* confers plant to salt and freezing tolerance in Arabidopsis, while transgenic soybean plants overexpressing *GmMYB84* have a superior ability to withstand drought stress [20,21].

Sweet potato counts among the most widely staple crops cultivated worldwide [22,23]. Due to its strong ecological adaptability, a high yield potential, and excellent nutritional value, sweet potato has become an indispensable food crop, especially in developing country [24]. Although sweet potato has many unique properties, its normal growth can also be affected by adverse conditions, such as freezing, drought, and salt stress [25]. Up to now, several genes involved in drought or salinity have been isolated and characterized in sweet potato, including *IbLCYB2*, *IbC3H18*, *IbGTAT24*, *IbWRKY2*, *ItfWRKY70*, *IbMIPS1*, *IbMYB116*, etc. [25,26,27,28,29,30,31]. Although the functions of hundreds of TFs in stress response have been extensively examined in sweet potato, the role of R2R3-MYB TFs in salt tolerance and drought resistance in sweet potato remains unclear.

In this study, a novel R2R3-MYB gene *IbMYB48* was cloned from the sweet potato line JS6-5. Overexpression of *IbMYB48* gene in Arabidopsis enhanced salt and drought tolerance. The levels of ABA, jasmonic acid (JA), proline and superoxide dismutase (SOD) activities were significantly higher in transgenic plants under the salt and drought stress treatment, while the contents of malondialdehyde (MDA) and H_2_O_2_ were markedly lower. Overexpression of *IbMYB48* up-regulated genes involved in ABA biosynthetic pathway, JA biosynthesis and signaling pathway and reactive oxygen species (ROS) scavenging system, thereby improving salt and drought resistance in transgenic Arabidopsis.

## 2. Materials and Methods

### 2.1. Plant Materials and Growth Conditions

Sweet potato line JS6-5 was used for the cloning of *IbMYB48* gene, as well as JS6-5 was applied to the expression analysis of *IbMYB48* gene with multiple abiotic stress in this study. Arabidopsis (*Columbia*-0) plants were grown in a greenhouse maintained at 22 °C under 16/8 h day/night regime or on 1/2 MS medium at 22 °C on a 16/8 h day/night system.

### 2.2. Cloning and Sequence Analysis of IbMYB48 Gene and Its Promoter Region

The total procedure of RNA extraction experiment followed the manufacturer’s protocol of TransZol Up Kit (TransGen Biotech, Beijing, China), and first-strand cDNA was synthesized using PrimeScript^TM^ II 1st Strand cDNA Synthesis Kit (Takara, Dalian, China). According to the EST obtained in a previous study [32] and referring to the genomic data of Sweet potato GARDEN (http://sweetpotato-garden.kazusa.or.jp/, accessed on 13 January 2018), the cDNA sequence of *IbMYB48* was obtained. The PCR conditions were as follows: denaturation, 95 °C 20 s; annealing, 55 °C 20 s; and extension, 72 °C 60 s for 35 cycles. 

Genomic DNA extracted from JS6-5 was used to amplify the genomic sequence of *IbMYB48* using LA Taq enzyme (Takara, Dalian, China). The PCR conditions were as follows: denaturation, 95 °C 30 s; annealing, 55 °C 30 s; and extension, 72 °C 360 s for 32 cycles. We used the Genome Walking Kit (Clonetech, Palo Alto, CA, USA) to amplify the promoter sequence of the *IbMYB48* gene as described previously [33]. 

Multiple protein sequences of IbMYB48 were aligned by the DNAMAN software (Lynnon Biosoft, Quebec, Canada). The molecular weight and theoretical isoelectric point (pI) of IbMYB48 were estimated from ExPASy (http://web.expasy.org/compute_pi/, accessed on 3 January 2018). The phylogenic tree was constructed using MEGA 6.0 software with the neighbor-joining method. The *IbMYB48* promoter region was screened for *cis*-acting regulatory elements with Plant CARE (http://bioinformatics.psb.ugent.be/ webtools/plantcare/html/, accessed on 10 March 2020). The primers were listed in Supplementary Materials Table S1.

### 2.3. Expression Analysis of IbMYB48 in Sweet Potato

The transcript levels of *IbMYB48* in the roots, stems, and leaves, and under different stresses of JS6-5 plants were analyzed using quantitative real-time PCR (qRT-PCR). The roots of 4-week-old in vitro-grown plants were soaked in 1/2 Hoagland solution with 200 mM NaCl, 20% PEG6000, 100 μM MeJA, 100 μM ABA, 100 μM salicylic acid (SA) and 10 mM H_2_O_2_, respectively. The whole plants were sampled 0, 0.5 h, 1 h, 3 h, 6 h, 12 h, 24 h and 48 h after treatments and then analyzed for the expression of *IbMYB48*.

The RNA was extracted using the TransZol method mentioned above. The qRT-PCR assay conditions were 95 °C 5 s for denaturation, 60 °C 34 s for annealing and extension, and 45 cycles. The gene relative mRNA expression levels were measured with the primers IbMYB48-qPCR-F/R, and *IbActin* was used as the internal control. The genes relative expression level was counted with the comparative C_T_ method [34]. Results are indicated as means ± SE of biological replicates (*n* = 3). The primers were listed in Supplementary Materials Table S1.

### 2.4. Subcellular Localization of IbMYB48

The coding region of *IbMYB48* was amplified using PCR primers 83-IbMYB48-F/R listed in Table S1. *Pac*I/*Asc*I-digested plasmids pMDC83-green fluorescent protein (GFP) and pMDC83-IbMYB48-GFP were constructed and separately transformed into fresh onion epidermal cells by particle bombardment with a Gene Gun (Bio-Rad Laboratories, Hercules, CA, USA). The onion cells were observed with fluorescent microscope Nikon TE-2000E (Nikon, Tokyo, Japan) using an excitation wavelength of 488 nm. The primers were listed in Supplementary Materials Table S1.

### 2.5. Transcriptional Activation Activity Analysis of IbMYB48

The open reading frame (ORF) of *IbMYB48* and two truncated variants were cloned separately into the *Nde*I/*Sal*I-digested pGBKT7 vector (ClonTech, Beijing, China). The pGBKT7-Lam and pGBKT7-53 vectors were used as a negative and positive control, respectively. The fusion plasmids, negative control, and positive control were transformed into the yeast strain Y2HGold (ClonTech, Beijing, China), individually. The transformed yeast was subjected to selection on SD/-Trp and SD/-Trp/-His/3-AT/X-α-gal mediums to observe the growth of yeast at 30 °C for 3–5 days. The primers used for transcriptional activation activity analysis were listed in Supplementary Materials Table S1.

### 2.6. Vector Construction and Transformation of IbMYB48 into Arabidopsis

The coding sequence of *IbMYB48* was amplified by using primers IbMYB48-OE-F/R and then was inserted into the *Kpn*I/*Sal*I-digested pCAMBIA1300-GFP expressing vector under the control of the CaMV 35S promoter. The promoter sequence of *IbMYB48* was amplified via using 162-IbMYB48-F/R. This DNA fragment was then inserted into a binary vector pMDC162 and fused to a β-glucuronidase (*GUS*) reporter gene to create a recombinant transcription unit, *IbMYB48pro⸬GUS*. The pCAMBIA1300-IbMYB48-GFP (Figure S1) and pMDC162-*IbMYB48pro⸬GUS* vectors were introduced into Arabidopsis using *Agrobacterium*-mediated floral dip method, respectively [35].

Seeds were surface sterilized and grown on 1/2 MS medium containing 50 mg/L hygromycin for selection. Independent *IbMYB48* overexpression lines from homozygous in T_3_ generation were obtained. DNA was extracted from leaves and the presence of the hygromycin gene was detected by PCR. Three transgenic lines (L6, L8 and L12) with the higher expression of *IbMYB48* were selected for subsequent experiments. The sequences of the primers used were listed in Supplementary Materials Table S1.

### 2.7. Stress Treatments of IbMYB48 Transgenic Plants

The method of stress treatments of *IbMYB48* transgenic plants according to the methods described by Du et al. [36] and Kang et al. [37]. Firstly, 30 seeds of *IbMYB48* transgenic Arabidopsis and WT were germinated on 1/2 MS medium. Then, seedlings were cultured in 1/2 MS medium with 100 mM NaCl or 300 mM mannitol, respectively. After 7 days, the root length, fresh weight and germination rate of transgenic plants and WT plants were measured by the method of Du et al. [36]. 

Furthermore, transgenic plants and WT seedlings were grown in pots and regularly watered for 10 days. The plants were then watered with 100 mM NaCl or 300 mM mannitol every 2 days for 3 weeks. The drought treatment adopted the natural drought method for 3 weeks, and the plant growth status was observed after the re-watering treatment for 2 days. The contents of ABA, JA, proline, MDA, H_2_O_2_ and SOD were measured by the methods of Zhai et al. [28], Zhou et al. [26] and Kang et al. [37].

### 2.8. Histochemical GUS Staining

Indirect GUS histochemical staining of transgenic Arabidopsis plants containing *IbMYB48pro⸬GUS* fusion construct followed the previously described method [38,39,40]. The stained plants were photographed using a digital camera (Nikon D800).

### 2.9. Statistical Analysis

All the experiments were repeated three times individually. The data are presented as the mean ± SD. The results were analyzed using Student’s *t*-test in a two-tailed analysis. 

## 3. Results

### 3.1. IbMYB48 Is a Potential Candidate Gene Involved in the Regulation of Tolerance to Abiotic Stress

**Figure 1:**
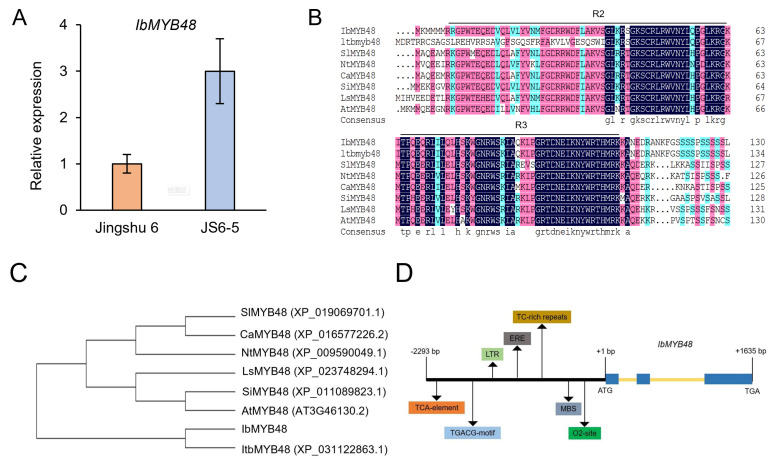
Sequence analysis of *IbMYB48*. (**A**) Expression level analysis of *IbMYB48* in storage root of Jingshu 6 and JS6-5. (**B**) Multiple sequence alignment of IbMYB48 and its closest orthologues from various species. The black lines represent the R2R3 domain of MYB transcription factor. (**C**) Phylogenetic analysis of MYB proteins from different plants. (**D**) An overview of *IbMYB48* promoter which contains different kinds of *cis*-acting elements, as well as the structure of the *IbMYB48* gene. Black arrows represent different *cis*-acting elements. Exon sequence is represented by blue boxes and introns are represented by yellow lines.

We previously conducted root transcriptomes of the sweetpotato variety Jingshu 6 and its mutant JS6-5 with high anthocyanin and increased tolerance to salt and drought stresses by high-throughput RNA sequencing [32]. Among these differential genes, the expression of a transcription factor gene, *IbMYB48* was obviously up-regulated in JS6-5 [32]. This trend was further supported by qRT-PCR analysis (Figure 1A). Therefore, *IbMYB48* gene was selected for subsequent analysis.

The full-length of *IbMYB48* cDNA was isolated from mutant line JS6-5. The ORF of *IbMYB48* consists of 801 bp, encoding a protein of 266 amino acids with a calculated molecular mass of 30.9 kDa and a calculated isoelectric point of 8.73. The IbMYB48 belongs to R2R3-MYB transcription factor subgroup (Figure 1B). Based on multiple sequence alignment, this protein shared a high sequence identity with MYB48 proteins in *Ipomoea triloba* (XP_031122863.1, 79.35%), *Capsicum annuum* (XP_016577226.2, 47.10%), *Nicotiana tomentosiformis* (XP_009590049.1, 46.38%), *Sesamum indicum* (XP_011089823.1, 45.65%), *Solanum lycopersicum* (XP_019069701.1, 44.57%), *Lactuca sativa* (XP_023748294.1, 43.12%), *Arabidopsis thaliana* (AT3G46130.2, 38.97%). Phylogenetic analysis showed that IbMYB48 has a close relationship with that of *Ipomoea triloba* (Figure 1C).

Next, the promoter region of the *IbMYB48* gene was cloned from the JS6-5 genomic DNA by the Genome Walking method [41]. The size of 2293 bp promoter contained many kinds of regulatory elements, such as TCA (*cis*-acting element involved in salicylic acid responsiveness), TGACG (*cis*-acting regulatory element involved in the MeJA-responsiveness), LTR (*cis*-acting element involved in low-temperature responsiveness), ERE (ethylene-responsive element), MBS (MYB binding site involved in drought-inducibility) and O2-site (*cis*-acting regulatory element involved in zein metabolism regulation) motif, of which we mainly focused on elements associated with abiotic stress resistance (Figure 1D). Moreover, The *IbMYB48* gene consists of a total of 3 exons and 2 introns through comparison of ORF and genomic sequence of *IbMYB48* (Figure 1D).

### 3.2. Expression Level of IbMYB48 Responses to Salt or Drought Treatments

**Figure 2:**
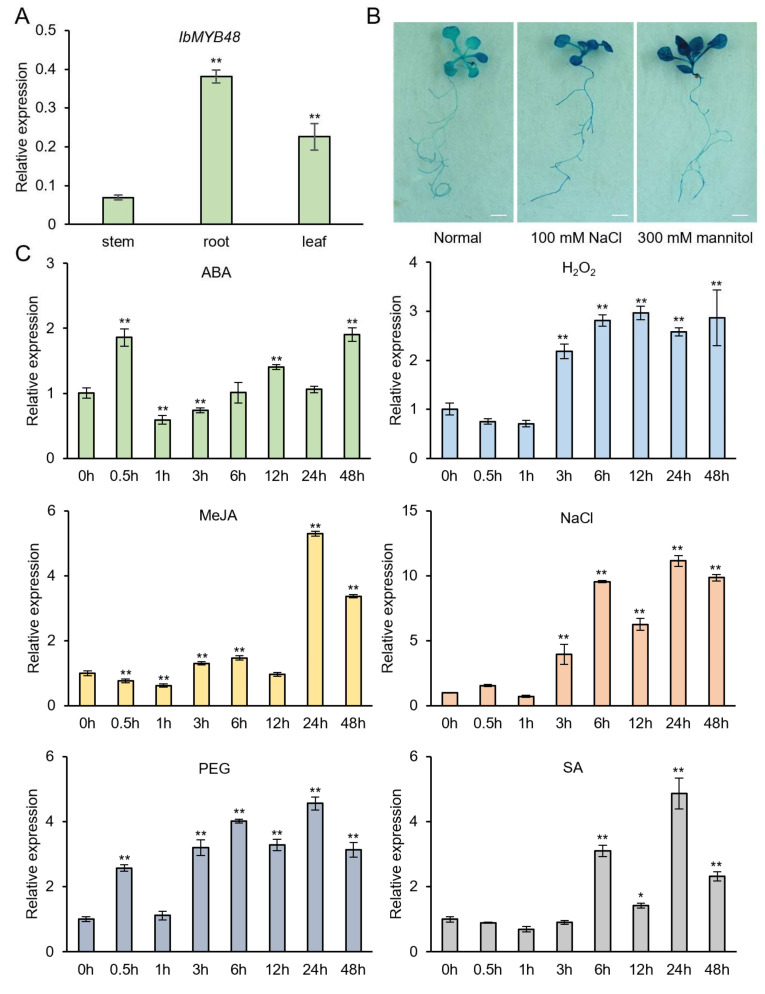
Expression analysis of *IbMYB48*. (**A**) Tissue-specific expression of *IbMYB48* in the stem, root, and leaf of JS6-5 plants. (**B**) Histochemical staining of GUS activity in *IbMYB48pro⸬GUS* transgenic Arabidopsis plants subjected to salt stress. Bars = 3 mm. (**C**) Expression analysis of *IbMYB48* in whole plants of JS6-5 in response to 100 μM ABA, 10 mM H_2_O_2_, 100 μM MeJA, 200 mM NaCl, 20% PEG6000 and 100 μM SA, respectively. The *IbActin* was used as the reference. Error bars reflect ± SE scores (*n* = 3); Asterisk (*) and (**) indicate a significant difference at the level *p* < 0.05, *p* < 0.01, individually.

To gain more insight into the biological function of *IbMYB48*, the expression profiling of *IbMYB48* in vitro-grown plants of JS6-5 were examined by qRT-PCR. Our results revealed that *IbMYB48* was widely detected in different tissues such as stem, root, and leaf, but it was predominantly expressed in leaf and root (Figure 2A).

To investigate whether *IbMYB48* is involved in salt or drought stress in sweet potato, the *IbMYB48pro⸬GUS* fusion plasmid was transformed into Arabidopsis. Two weeks old transgenic Arabidopsis plants were treated with 100 mM NaCl and 300 mM mannitol for 12 h and stained before and after treatment. The results showed that promoter region from *IbMYB48* was sufficient to confer *GUS* expression in whole plants. We observed stronger GUS staining in both roots and leaves of *IbMYB48pro⸬GUS* lines after NaCl and mannitol treatments, indicating that the *IbMYB48* promoter contains *cis*-regulatory elements responding to NaCl and mannitol (Figure 2B).

To further analyze its potential function, the expression of *IbMYB48* was analyzed under different stress conditions, including 100 μM ABA, 10 mM H_2_O_2__, _100 μM MeJA, 200 mM NaCl, 20% PEG6000, and 100 μM SA for 0, 0.5 h, 1 h, 3 h, 6 h, 12 h, 24 h and 48 h, respectively. These results showed that the expression of *IbWRKY48* was significantly induced by ABA, H_2_O_2_, MeJA, NaCl, PEG, and SA, and peaked at 48 h with 1.9-fold, 12 h with 3.0-fold, 24 h with 5.3-fold, 24 h with 11.0-fold, 24 h with 4.5-fold, and 24 h with 4.9-fold, respectively (Figure 2C). 

### 3.3. IbMYB48 Is Localized in the Nucleus

**Figure 3:**
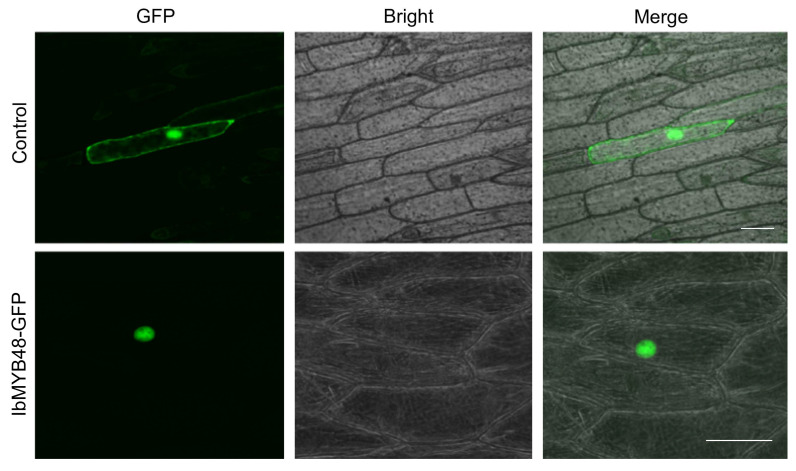
Transient expression and localization analysis of IbMYB48-GFP in onion epidermal cells. Specimens were observed using fluorescent microscope Nikon TE-2000E with 488 nm excitation wavelength laser. Bars = 30 μm.

To explore the subcellular localization of the IbMYB48, we performed a transient expression experiment of *IbMYB48* in onion epidermal cells. The C terminus of *IbMYB48* was fused with *GFP* under the control of the CaMV 35S promoter, and the construct was introduced into onion epidermal cells using gene gun method [42]. In the cells expressing GFP alone, the signal was detected in the cytoplasm and nucleus. In contrast, the green fluorescence signal from the IbMYB48-GFP fusion protein was mainly detected in the nucleus, indicating that IbMYB48 is a nucleus protein (Figure 3).

### 3.4. IbMYB48 Possesses Transcriptional Activation Activity

**Figure 4:**
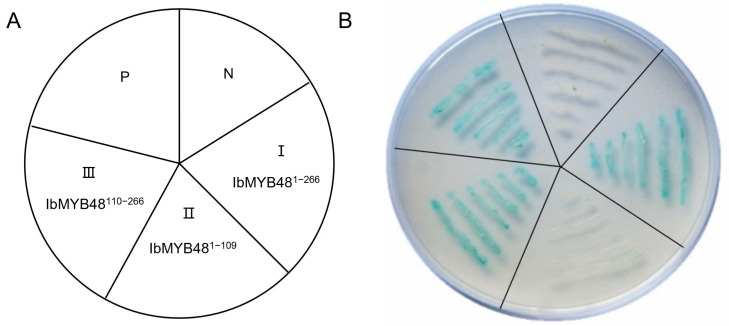
Experiment of IbMYB48 transactivation activity in yeast using full-length as well as truncated forms. (**A**) The pGBKT7-53 (P) and pGBKT7-Lam (N) were used as positive and negative control, respectively. Part I, Part II, and Part III represents the pGBKT7-IbMYB48^1−266^, pGBKT7-IbMYB48^1−109^ and pGBKT7-IbMYB48^110−266^ were transformed into Y2HGold, individually. (**B**) The transformed yeast cells were grown in SD/-Trp/-His/3-AT medium with X-α-gal.

To analyze whether IbMYB48 can serve as a transcriptional activator and which region of IbMYB48 has the transcriptional activity, full-length and different truncation of IbMYB48 construct vectors (pGBKT7-IbMYB48^1^^–109^, pGBKT7-IbMYB48^110^^–266^, pGBKT7-IbMYB48^1–266^) were constructed for transactivation analysis in yeast. The resulting plasmids, pGBKT7-Lam (negative control) and pGBKT7-53 (positive control) were then transformed into yeast strain Y2HGold, respectively (Figure 4A). 

Yeast cells carrying any one of the five vectors grew well on SD/-Trp medium. Yeast cells containing pGBKT7-IbMYB48^110−266^, pGBKT7-IbMYB48^1−266^ and the positive control grew properly on SD/-Trp/-His/3-AT/X-α-gal medium displaying α-galactosidase activity, whereas the yeast cell containing pGBKT7-IbMYB48^1−109^ or negative control did not grow (Figure 4B). These results demonstrate that IbMYB48 is a nuclear protein and C-terminal region of IbMYB48 is responsible for its transcriptional activation activity.

### 3.5. Expression of IbMYB48 Enhances Salt and Drought Tolerance in Arabidopsis

**Figure 5:**
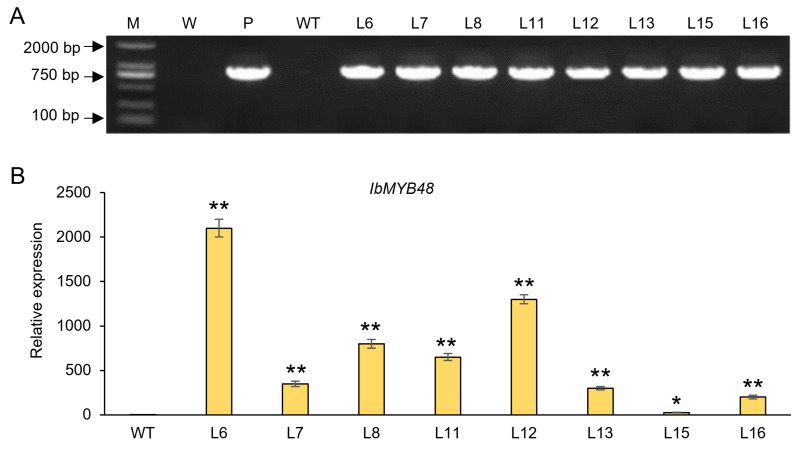
Identification of *IbMYB48*-overexpression transgenic plants. (**A**) Identification of transgenic lines by PCR. M, DL2000 DNA maker; W, water as a negative control; P, plasmid pCAMBIA1300-IbMYB48-GFP as positive control; WT, wild plant as negative control. (**B**) The expression level of *IbMYB48* in different transgenic lines by qRT-PCR. Asterisk (*) and (**) indicate a significant difference at the level *p* < 0.05, *p* < 0.01, individually.

To further analyze the biological function of *IbMYB48* in plants, we next generated transgenic plants carrying *IbMYB48* under the control of the CaMV 35S promoter. A total of 8 positive T_3_ transgenic lines were obtained (Figure 5A). The mRNA expression level of *IbMYB48* in different lines was analyzed by qRT-PCR (Figure 5B). Three independent lines (L6, L8, and L12) with the high expression level were chosen for subsequent physiology experiments. 

**Figure 6:**
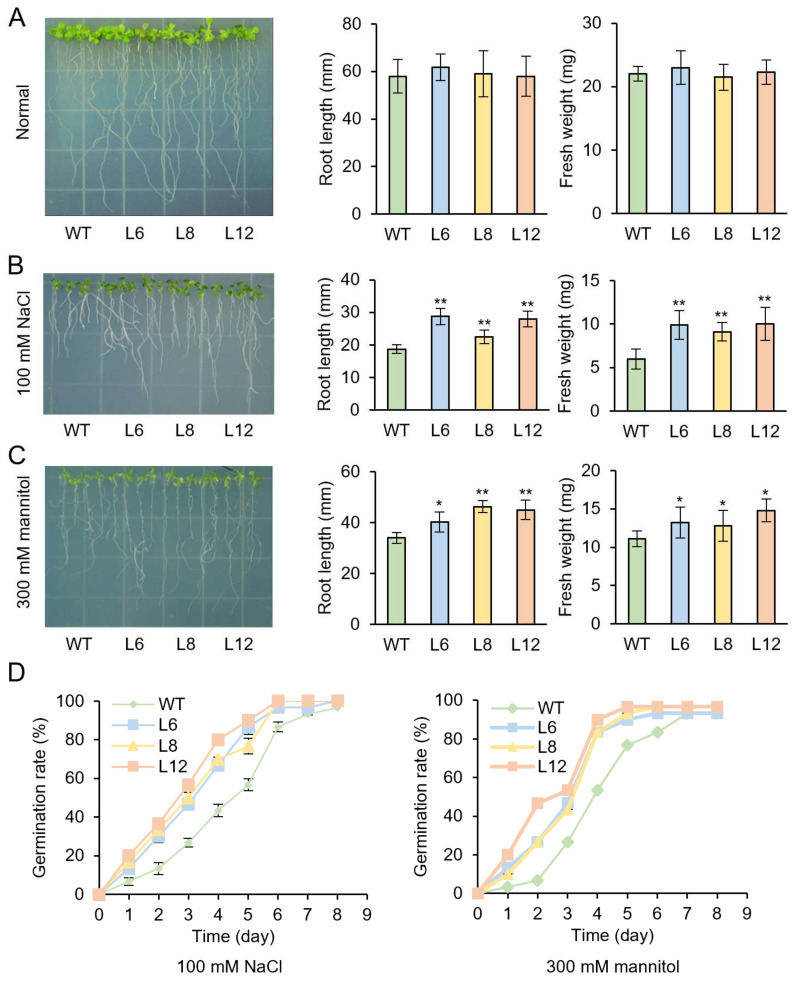
Response of transgenic and WT seedlings under salt and drought stresses. (**A**–**C**) Root length and fresh weight of WT and transgenic lines cultivated separately on 1/2 MS medium with normal (**A**), 100 mM NaCl (**B**) or 300 mM mannitol (**C**) for 7 days. (**D**) The germination rate of transgenic and WT seeds, which were cultured on 1/2 MS medium with 100 mM NaCl or 300 mM mannitol, respectively. Asterisk (*) and (**) indicate a significant difference from the WT at values of *p* < 0.05 and *p* < 0.01, individually, by Student’s *t*-test.

Then, transgenic plants and WT seedlings were treated with different stress conditions. T_3_ transgenic lines L6, L8, L12 and WT seeds were cultured on 1/2 MS medium which contains 100 mM NaCl or 300 mM mannitol for 7 days, respectively. There were no phenotypic differences between the WT and transgenic plants under normal conditions (Figure 6A). However, the growth status of WT was inferior to the transgenic lines under NaCl or mannitol treatments. We assessed plant tolerance to abiotic stress by measuring the length and fresh weight of roots. The root length of transgenic plants was longer than WT and the fresh weight of transgenic plants was higher than WT under both salt and mannitol treatments (Figure 6B,C).

During the seed’s germination stage, the germination rate of the WT seeds was markedly decreased, while *IbMYB48*-overexpression transgenic plants exhibited higher germination rate compared with WT under NaCl and mannitol stresses. These results indicated that overexpression of *IbMYB48* could enhance the resistance to salt and drought stresses (Figure 6D).

**Figure 7:**
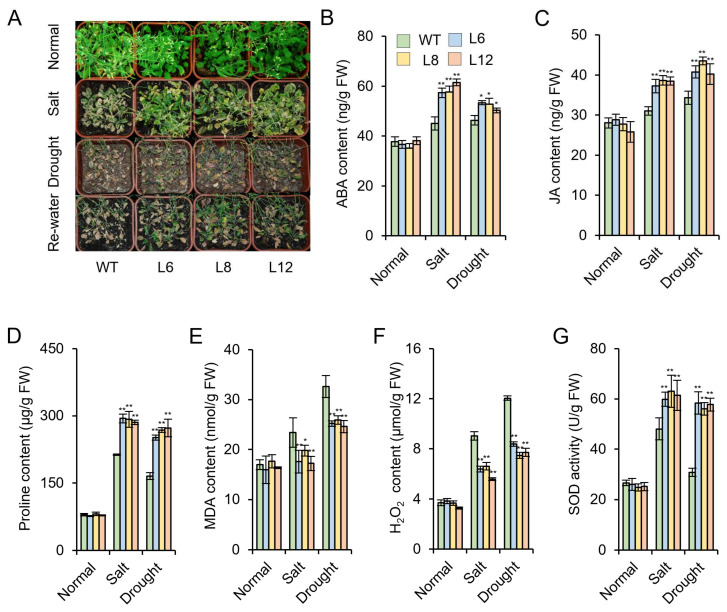
Responses of transgenic lines and WT grown in pots under salt and drought stresses. (**A**) The transgenic lines and WT grown in 1/2 Hoagland solution with 300 mM NaCl. Performance of transgenic lines and WT treated with soil drought stress without water for 3 weeks and then recovered for 2 days. Bar = 2 cm. (**B**–**G**) ABA (**B**), JA (**C**), proline (**D**), MDA (**E**), and H_2_O_2_ (**F**) contents and SOD activity (**G**) in the transgenic Arabidopsis plants and WT under salt and drought stress. Values are shown as mean ± SE (*n* = 3); Asterisk (*) and (**) indicate that there is a significant difference at *p* < 0.05 and *p* < 0.01, individually.

To further verify *IbMYB48* could contribute to salt resistance, the above transgenic lines were grown in potting soil mixture under normal condition for 10 days; And then, these lines were treated with 1/2 Hoagland solution supplemented with 300 mM NaCl for 3 weeks. The leaves of the WT exhibited chlorosis and wilted, while the leaves of the transgenic lines remained green and the overall growth performances was better than WT under salt stress. Under drought treatment, most of the WT wilted and died, while the majority of the transgenic lines survived and showed relatively good growth (Figure 7A). Taken together, these morphological changes indicated that transgenic lines have better growth under salt and drought stresses.

To explore the potential mechanism of *IbMYB48* in stress response, several physiological indexes related to stress tolerance were evaluated. The contents of ABA, JA and proline were considerably increased in transgenic lines compared to the WT, and the contents of MDA and H_2_O_2_ were significantly decreased in the transgenic plants after salt and drought treatments (Figure 7B–F). In addition, SOD activity was increased by 28% and 86% in transgenic plants under salt and drought treatments, respectively (Figure 7G). All these findings indicated that transgenic lines show decreased sensitivity to salt and drought stresses by regulating the plant hormone levels and osmotic balance.

### 3.6. Expression of the Stress-Related Genes Are Up-regulated in Transgenic Arabidopsis Plants

**Figure 8:**
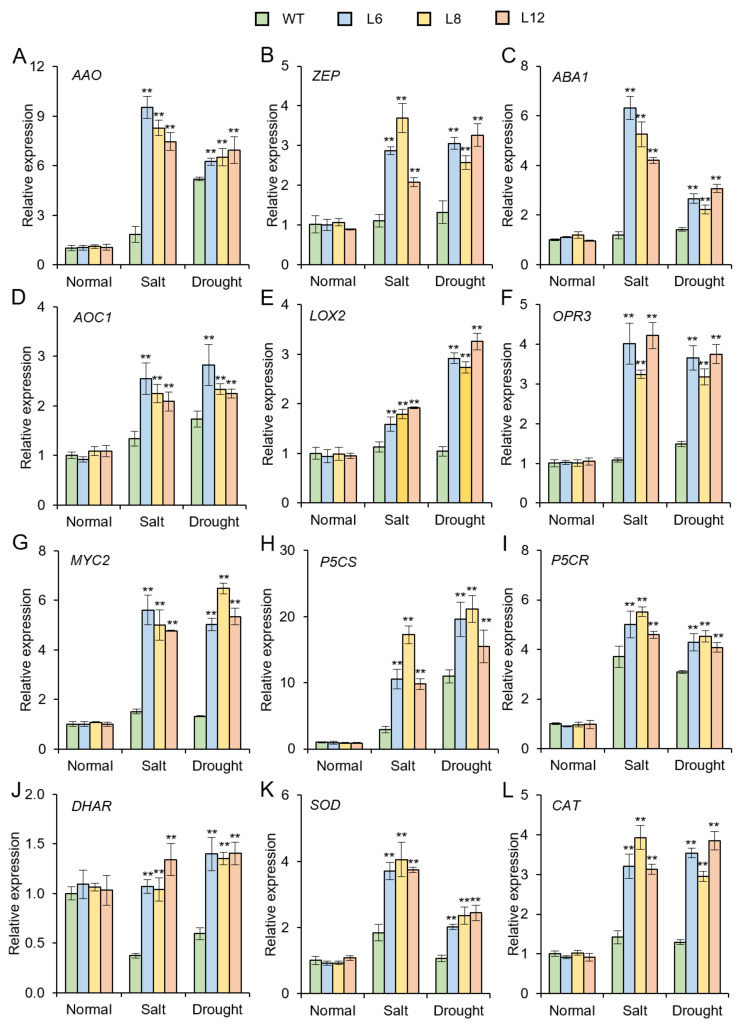
The expression profile of stress-related genes in transgenic lines and WT under salt and drought treatments for 2 weeks. The aerial parts of transgenic and WT plants were collected to extract total RNA. qRT-PCR analysis of the expression of ABA biosynthetic genes (**A**–**C**), JA biosynthesis and signaling pathway genes (**D**–**G**), proline biosynthetic genes (**H**,**I**) and ROS-scavenging genes (**J**–**L**). Values are indicated as means ± SE (*n* = 3); Asterisk (**) indicates that there is a significant difference at *p* < 0.01, individually. *AAO*, ABA-aldehyde oxidase; *ZEP*, zeaxanthin epoxidase; *ABA1*, ABA-deficient 1; *AOC1*, allene oxide cyclase 1; *LOX2*, 13-lipoxygenase 2; *OPR3*, 12-oxophytodienoate reductase 3; *P5CS*, pyrroline-5-carboxylate synthase; *P5CR*, pyrroline-5-carboxylate reductase; *DHAR*, dehydroascorbate reductase; *SOD*, superoxide dismutase; *CAT*, catalase.

To further investigate the molecular mechanism underlying the *IbMYB48* involved in salt and drought resistance, the expression levels of stress-responsive genes were investigated. Under salt and drought treatments, overexpression of *IbMYB48* up-regulated the genes involved in ABA biosynthesis (*AAO*, *ZEP* and *ABA1*), JA biosynthesis and signaling pathway (*AOC1*, *LOX2*, *OPR3* and *MYC2*), proline biosynthesis (*P5CS* and *P5CR*), and the reactive oxygen species (ROS) scavenging (*DHAR*, *SOD* and *CAT*) in transgenic lines compared to WT (Figure 8).

## 4. Discussion

TFs act as principal modulators of plant growth progresses. Hence, they are expected to be superb candidates for improving important traits in crops [43]. MYB transcription factors represent one of the largest protein family in plants [44]. A growing number of studies have shown that the R2R3-MYB presumably has various functions in the regulation of different abiotic stress responses. For instance, it is demonstrated that *AtMYB2* and *AtMYB60* are engaged in drought tolerance stress in plants [14,15]. *AtMYB44* and *AtMYB2* respond to salt stress at the transcript level and overexpression of *AtMYB44* and *AtMYB2* could enhance the resistance of transgenic Arabidopsis plants to salt stress [14,17]. Heterologous expression of *GmMYB81* in Arabidopsis can intensify salt and drought tolerance during seed germination [45]. Several R2R3-MYB genes, such as rice *OsMYB91*, apple *MdSIMYB1* and wheat *TaMYB33*, can increase salt and drought tolerance of transgenic plants [46,47,48]. However, few MYB family members have been studied in sweet potato under abiotic stress conditions. 

Here, we cloned a MYB member obtained from the transcriptome database. The R2R3-MYB functional domain has been identified in IbMYB48 (Figure 1B). IbMYB48 possesses transcriptional activation activity, implying that IbMYB48 may act as an activator to affect the expression of downstream genes (Figure 4B). In further experiments, *IbMYB48* is proved to be induced by ABA, H_2_O_2_, MeJA, NaCl, PEG6000 and SA treatments. Overexpression of *IbMYB48* confers salt and drought tolerance in Arabidopsis (Figure 2C and Figure 7A). 

In Arabidopsis, *AtMYB60* transcript is primarily expressed in guard cells [49]. Mutation of this gene leads to guard-cell-specific defects without affecting other developmental and physiological processes [49]. *AtMYB52* is much more abundantly expressed in roots and siliques compared to flowers and leaves [50]. In rice, *OsMYB48-1* is expressed most abundantly in roots at both seedling stage and reproductive stage but expressed at a low level in the sheath at seedling stage [51]. The highest expression level of *OsMYB2* is measured in leaves, which was followed by roots and shoots [52]. In soybean, *GsMYB15* is mainly detected in the roots, leaves and stems of transgenic plants and was a notably strong expression in the pods and flowers [53]. In the present research, tissue expression pattern analysis exhibited that *IbMYB48* had higher expression levels in leaf and root (Figure 2A). Compared to other MYB genes in the above species, differential tissue expression patterns of *IbMYB48* implied that it might have different functions. Furthermore, the relevant physiological parameters of *IbMYB48*-overexpression transgenic Arabidopsis under salt and drought stresses are consistent with those of *ZmMYB3R*, *ZmMYB48* and *FtMYB9*, which were described to be engaged in salt or drought tolerance in plants. The *ZmMYB3R-*overexpression Arabidopsis plants show increased activities of the antioxidant enzymes CAT, POD and SOD after salt and drought treatments compared to WT plants [54]. The contents of proline and MDA are elevated in both *ZmMYB48*-overexpression Arabidopsis plants and WT under drought stress; however, the transgenic plants have markedly higher proline content compared to WT as well as significantly lower MDA content than WT [55]. When plants are subjected to salt and drought treatments, the MDA content of *FtMYB9*-overexpression Arabidopsis plants are lower than WT [56]. In addition, the proline contents of *FtMYB9* transgenic plants are shown to be higher than those of WT during normal and abiotic stress conditions [56]. In the present research, we found that the contents of proline and SOD activities were significantly increased, and the contents of MDA and H_2_O_2_ were significantly decreased in transgenic plants under salt and drought stresses, suggesting that transgenic plants maintain intracellular osmotic balance and cell membrane integrity (Figure 7D–G).

In plants, ABA biosynthetic pathway related to salt and drought stress have been elucidated [57]. ABA is biosynthesized *de novo* from a C_40_ carotenoid precursor β-carotene by the oxidative cleavage of neoxanthin as well as a two-step conversion of xanthoxin to ABA via ABA-aldehyde [57,58]. There are some key enzymes that are essential for this series of synthetic reactions, like ZEP, 9-*cis*-epoxycarotenoid dioxygenase (NCED), AAO, molybdenum cofactor sulfurase (MCSU) [58]. *ABA1* encodes a ZEP that has an important role in ABA biosynthesis [59]. When plants are exposed to salt stress, the ABA concentration is distinctly increased [60]. Meantime, ABA can increase the proline accumulation in plants [61]. Some identified MYB proteins could response to abiotic stress through ABA biosynthetic pathways, such as OsMYB48-1, OsMYB91, ZmMYB3R and TaMYB33. The reaction catalyzed by NCED to produce xanthoxin from neoxanthin in plastid is commonly recognized to be the rate-limiting step in the ABA biosynthetic processes [62,63]. Finally, ABA2 and AAO3 convert xanthoxin into ABA in the cytoplasm [64,65]. The expression levels of *OsNCED4* and *OsNCED5* were higher in *OsMYB48-1* overexpression lines than WT under drought stress [51]. Salt-inducible OsMYB91 confers tolerance to salt in transgenic plants, and ABA synthesis genes *OsPDS*, *OsZDS*, *OsZEP* and *OsNCED4* were up-regulated in the *OsMYB91* overexpression plants compared to WT [47]. In *TaMYB33* overexpression lines, the expression of ABA synthesis gene *AtAAO3* is remarkably up-regulated under NaCl and PEG treatments [48]. Interestingly, the ABA content and expression levels of ABA biosynthetic genes *ABA1*, *ABA2* and *NCED3* are higher in *ZmMYB3R*-overexpression transgenic lines than in WT plants after salt and drought treatments [54]. Similarly, in the present research, it was demonstrated that the contents of ABA in transgenic plants are higher than the WT under salt and drought stresses (Figure 7B). Overexpression of *IbMYB48* up-regulated the genes associated with the ABA biosynthetic pathway (*AAO*, *ZEP, ABA1*) in overexpression lines compared with WT under salt and drought treatments (Figure 8A–C). these results indicate that *IbMYB48* may participate in ABA biosynthetic pathway along with additional factors to enhance the abiotic stress resistance. It is generally considered that plant salt and drought-responsive genes are mainly involved in both ABA-independent and ABA-dependent pathways [1]. RD29A and ERD1 play an important role in ABA-independent pathway [66,67]. In wheat, the expression level of *RD29A* and *ERD1* is significantly up-regulated in the *TaMYB30-B*-overexpression transgenic plants [19]. In *GmMYB118*-overexpression plants, the expression levels of *DREB2A*, *RD29A* are induced under drought stress, indicating that *GmMYB118* could improve plant tolerance to drought stress by activating both the ABA-independent and ABA-dependent pathway genes [68].

JA is a compound derived from lipids that function as a signal molecule during plant stress response and development [69]. In Arabidopsis, there are numerous enzymes involved in the JAs biosynthesis have been extensively characterized, such as phospholipases (PLA), LOX, allene oxide synthase (AOS), AOC, OPR3, OPC-8:0 CoA ligase 1 (OPCL1) and thioesterase (TE) [69,70,71]. In the present research, we demonstrated that the expression of *IbMYB48* was the highest at 24 h when the sweet potato mutant JS6-5 were treated with 10 mM MeJA (Figure 2). The JA content was increased under salt and drought stresses in *IbMYB48*-overexpression transgenic plants compared with WT (Figure 7C). Similarly, *ThMYB9* was strongly induced by JA treatment, and its expression reached the maximum at 72 h [72]. *JcMYB2* expression level is gradually increased as well as also reached a peak level at 6 h under MeJA treatment, then dramatically decreased and back to the primal level [73]. Additionally, MeJA could induce *GUS* expression in *JcMYB2pro⸬GUS* transgenic plants via GUS histochemical staining [73]. In the present research, under salt and drought stresses, the JA biosynthesis and signaling genes *LOX2*, *OPR3, AOC1* and *MYC2* were up-regulated in the *IbMYB48*-overexpression transgenic plants compared with WT (Figure 8D–G). These results imply that the genes of JA biosynthesis pathway and JA signaling transduction are activated by *IbMYB48* and improve abiotic stress tolerance of *IbMYB48*-overexpression transgenic plants. 

Proline is one of the osmotic stress regulation substances that respond to cell dehydration [74]. Under drought stress, plants can accumulate more proline in response to stress [75]. In the present research, the contents of proline were considerably increased in transgenic lines under salt and drought treatments (Figure 7D). The expression of proline synthase genes *P5CS* and *P5CR* was significantly increased in transgenic plants compared with WT after salt and drought treatments (Figure 8H,I), which could improve the intracellular osmotic balance and cell membrane integrity of the transgenic plants, thereby enhancing the stress resistance of transgenic plants.

ROS plays a double role in response to abiotic stresses as toxic by-products of stress metabolism, as well as important signal transduction molecules in plants [76]. Singlet oxygen (^1^O_2_), superoxide (O_2_^−^), H_2_O_2_ and hydroxyl radicals (·OH) are one of the main forms of ROS in plants [77]. ROS accumulation mainly depends on the dynamic balance between ROS production and ROS scavenging under salt and drought stresses [78]. It is imperative for plants to control ROS toxicity effects through ROS scavenge system. SOD and CAT were essential for ROS-scavenging mechanism [76,77]. In the present research, SOD activity and H_2_O_2_ content were significantly increased and decreased in *IbMYB48*-overexpression transgenic plants than those in WT under salt and drought stresses, respectively (Figure 7F,G). The expression of genes encoding *DHAR, SOD*, and *CAT*, members of the ROS scavenging system, was all increased in transgenic plants compared with WT (Figure 8J–L). These results indicated that overexpression of *IbMYB48* activates the genes of ROS scavenging system under salt and drought stresses and improves the salt and drought tolerance.

**Figure 9:**
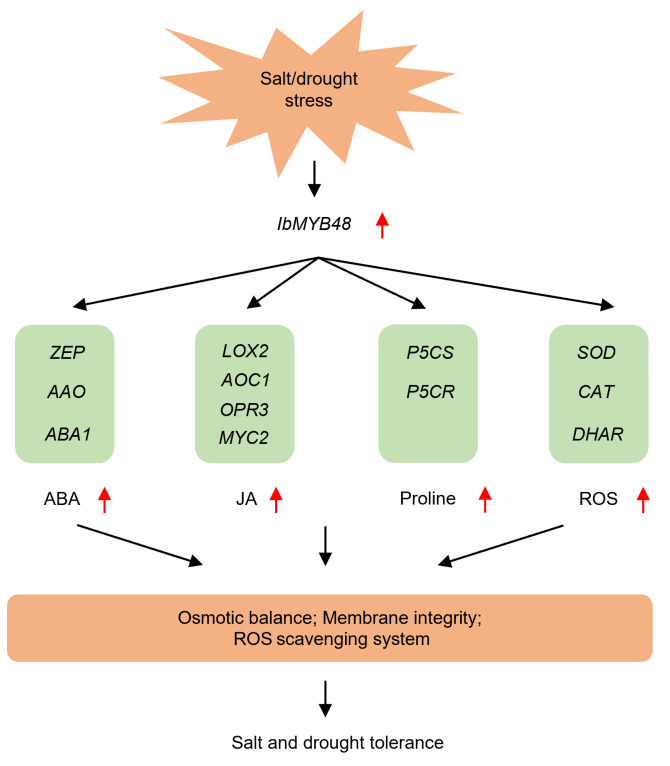
The proposed regulatory network model of *IbMYB48* conferring salt and drought tolerance. Under salt or drought stress, the expression level of *IbMYB48* gene is induced. Elevated expression of *IbMYB48* up-regulates the genes involved in the ABA, JA and proline biosynthetic or signaling pathways. As a result, the contents of ABA, JA, and proline are elevated, meanwhile, the ROS scavenging system is activated, thus improving salt and drought resistance in plants through maintaining osmotic balance and membrane integrity.

Taken together, these results reveal that the ABA biosynthetic pathway, JA biosynthesis and signaling pathway and ROS-scavenging system are activated by overexpression of *IbMYB48*, thus improving abiotic stress tolerance of transgenic plants (Figure 9). However, *IbMYB48* is only heterologously expressed in Arabidopsis for correlated functional validation. In the future, we will further investigate the potential function of *IbMYB48* in salt and drought tolerance by using gene-editing technology.

## 5. Conclusions

In conclusion, we cloned and characterized *IbMYB48* from sweet potato mutant line JS6-5. IbMYB48 was found to be a nuclear-protein, consistent with functioning as a TF. Self-transcriptional activation domain of IbMYB48 located in the C-terminal region. Ectopic expression of *IbMYB48* in Arabidopsis results in increased ABA, JA, proline contents and SOD activity, indicating that *IbMYB48* positively regulates tolerance to salt and drought stresses. Our results suggest *IbMYB48* may be a candidate gene for improving plant tolerance to abiotic stress.

## Supplementary Materials

The following supporting information can be downloaded at: https://www.mdpi.com/article/10.3390/genes13101883/s1, Table S1: The primers used in this study. Figure S1: The pCAMBIA1300-IbMYB48-GFP vector used in this study.
