# Supplementary material for: Expression of the Sweet Potato MYB Transcription Factor IbMYB48 Confers Salt and Drought Tolerance in Arabidopsis
Source: Genes (Basel). 2022 Oct 17;13(10):1883. doi: 10.3390/genes13101883 (PMC9602379; doi:10.3390/genes13101883)
Supplement: Supplementary file 1 [file genes-13-01883-s001.zip › genes-1940965-supplementary.pdf]

Table S1. The primers used in this study.

Primer name	Primer sequence
IbMYB48 GW-1	CTATTGCCCCATTTGGAGTGGAGTT
IbMYB48 GW-2	TTCAACCCTACCAAAAGCATGTGAA
IbMYB48 G-F	ATGAAGATGATGATGATGAGGAGA
IbMYB48 G-R	ATTATATTGTGCATAATCTTGGTTGT
IbMYB48 ORF-F	ATGAAGATGATGATGATGAGGAGA
IbMYB48 ORF-R	ATTATATTGTGCATAATCTTGGTTGT
IbMYB48 Pro-F	TGCCTTAGAGACACCATGGACT
IbMYB48 Pro-R	CATCTCTTCCTGTGCCATTTTC
IbActin-F	AGCAGCATGAAGATTAAGGTTGTAGCAC
IbActin-R	TGGAAAATTAGAAGCACTTCCTGTGAAC
IbMYB48-qPCR-F	ACCAGGACGAACCGACAAC
IbMYB48-qPCR-R	AGGAAGAGGAATTATTGGAGAGTG
83-IbMYB48-F	CCTTAATTAA ATGAAGATGATGATGATGAGGAGA
83-IbMYB48-R	AGGCGCGCCAATTATATTGTGCATAATCTTGGTTG
162-IbMYB48-F	CCTTAATTAATGCCTTAGAGACACCATGGACT
162-IbMYB48-R	AGGCGCGCCCATCTCTTCCTGTGCCATTTTC
BD1-109-F	GGAATTCCATATGATGAAGATGATGATGATGAGGAGAG
BD1-109-R	ACGCGTCGACTCACTTTTTCCTCATGTGGGTT
BD110-266-F	GGAATTCCATATG GCTAACGAAGACAGAGCCAAC
BD110-266-R	ACGCGTCGACTCAATTATATTGTGCATAATCTTGG
BD1-266-F	GGAATTCCATATGATGAAGATGATGATGATGAGGAGA
BD1-266-R	ACGCGTCGACTCAATTATATTGTGCATAATCTTGG
IbMYB48-OE-F	GGGGTACCATGAAGATGATGATGATGAGGAGA
IbMYB48-OE-R	ACGCGTCGACATTATATTGTGCATAATCTTGGTTGT
Actin-F	GCACCCTGTTCTTCTTACCGA
Actin-R	AGTAAGGTCACGTCCAGCAAGG
ZEP-F	CGGAGCTTTCTTCTTGATGG
ZEP-R	TCGATTTCGGAGTTTTCCTG
ABA-F	TACTTGGGGTAAAGGGCGTG
ABA-R	CCAAGGACCCAGTCAAGCAT
AAO-F	CAACAGCCATGTTGATACCG
AAO-R	TCTTTGACCTGCACATCGAG
LOX2-F	CAAACCTCAGAAGACGATGTAAGG
LOX2-R	GACCTCTCGACCAAGTTATGCC
MYC2-F	CGGGTCTTAATTGGAATCTTGAC
MYC2-R	TTCGGTTATTGTGCTTGAGCTAC
AOC1-F	ATCTATCTCCATGACAACTCTCAACAA
AOC1-R	AGGAACCTGGACCGTTAGATGA
OPR3-F	GGCACAAGGGAACTCTAACGAG
OPR3-R	CTTGTTCATCTGAATAGATTCCAGG
P5CS-F	ATGATCTTATTTATGTTCTGC
P5CS-R	CACTATCTTCCGTCACTAT

P5CR-F	AGTTTAGCTTCACAGACCGTTC
P5CR-R	GCTCTGTGAGAGCTCGCGGCTTC
SOD-F	ATGAGAAGTTCTATGAAGAG
SOD-R	GTCTTTATGTAATCTGGT
CAT-F	GCAACTACCCCGAGTGGAAA
CAT-R	TGTTCAGAACCAAGCGACCA
DHAR-F	ATGGTCCTTTTATCGCCGGG
DHAR-R	GCCCATCCAGAGATCACACA

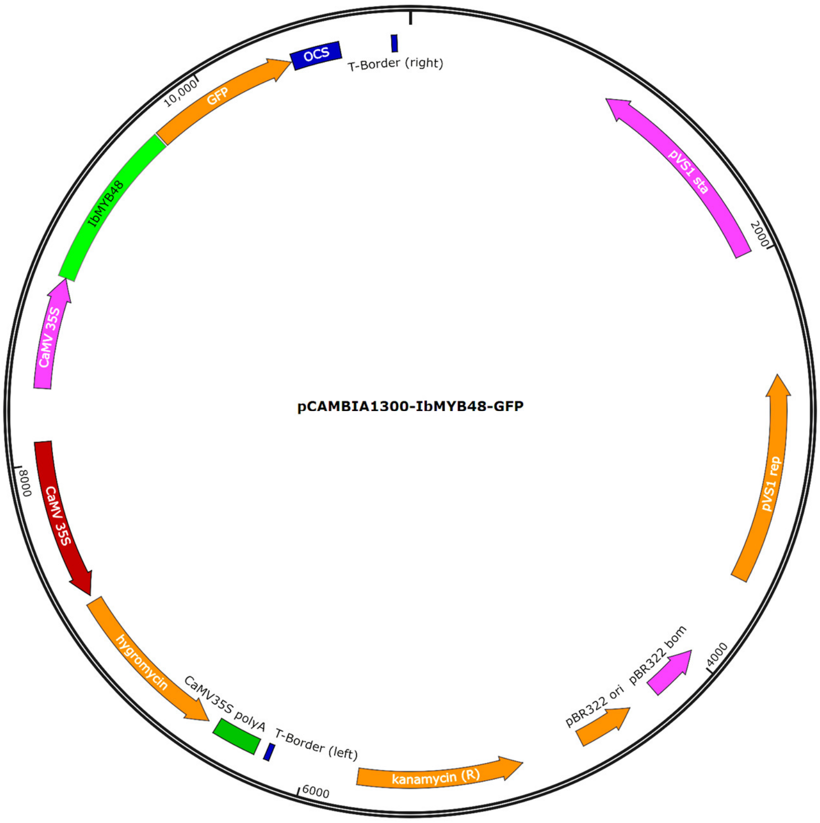

Figure S1. The pCAMBIA1300-IbMYB48-GFP vector used in this study.
